# Emergence of life in an inflationary universe

**DOI:** 10.1038/s41598-020-58060-0

**Published:** 2020-02-03

**Authors:** Tomonori Totani

**Affiliations:** 1 Department of Astronomy, School of Science, The University of Tokyo, Bunkyo-ku, Tokyo 113-0033 Japan; 2 Research Center for the Early Universe, School of Science, The University of Tokyo, Bunkyo-ku, Tokyo 113-0033 Japan

**Keywords:** RNA, Evolutionary theory, Astronomy and astrophysics, Cosmology, Exoplanets

## Abstract

Abiotic emergence of ordered information stored in the form of RNA is an important unresolved problem concerning the origin of life. A polymer longer than 40–100 nucleotides is necessary to expect a self-replicating activity, but the formation of such a long polymer having a correct nucleotide sequence by random reactions seems statistically unlikely. However, our universe, created by a single inflation event, likely includes more than 10^100^ Sun-like stars. If life can emerge at least once in such a large volume, it is not in contradiction with our observations of life on Earth, even if the expected number of abiogenesis events is negligibly small within the observable universe that contains only 10^22^ stars. Here, a quantitative relation is derived between the minimum RNA length *l*_min_ required to be the first biological polymer, and the universe size necessary to expect the formation of such a long and active RNA by randomly adding monomers. It is then shown that an active RNA can indeed be produced somewhere in an inflationary universe, giving a solution to the abiotic polymerization problem. On the other hand, *l*_min_ must be shorter than ~20 nucleotides for the abiogenesis probability close to unity on a terrestrial planet, but a self-replicating activity is not expected for such a short RNA. Therefore, if extraterrestrial organisms of a different origin from those on Earth are discovered in the future, it would imply an unknown mechanism at work to polymerize nucleotides much faster than random statistical processes.

## Introduction

In spite of recent rapid development of biology, chemistry, Earth science and astronomy, the origin of life (abiogenesis) is still a great mystery in science^1–5^. A prominent feature of life is the ordered information stored in DNA/RNA, and how such information appeared from abiotic processes is a crucial issue. The RNA world hypothesis^6–8^ postulates an early era when RNA played both the genetic and catalytic roles before the DNA-protein world came into being. This is widely accepted due to strong supporting evidence including catalytic activities of RNA, especially its central role in a ribosome. However, a more fundamental and unsolved problem is how an RNA polymer long enough to have a self-replicating RNA polymerase activity (i.e., RNA replicase ribozyme) emerged from prebiotic conditions and then triggered Darwinian evolution.

A key quantity is the minimum RNA length required to show a self-replicating ability. RNA molecules shorter than 25 nucleotides (nt) do not show a specified function, but there is a reasonable hope to find a functioning replicase ribozyme longer than 40–60 nt^8,9^. RNA polymerase ribozymes produced by *in vitro* experiments so far have a length longer than 100 nt^10–12^. Furthermore, formation of just a single long strand may not be sufficient to initiate an abiogenesis event. Instead a pair of identical strands may be necessary if one serves as a replicase ribozyme and the other as a template.

Polymerization of RNA in water is a thermodynamically uphill process, and hence reacting monomers need to be activated. Non-enzymatic reactions of adding activated monomers (e.g., imidazole-activated ribonucleotides) to an RNA oligomer have been experimentally studied^1,4,13^. Reactions at inorganic catalytic sites (e.g. surface of minerals such as montmorillonite clay) may be particularly efficient^14,15^. Some experiments yielded production of up to 40-mers of RNA^16,17^, which may be long enough to have some biological activities. However, these results have not been reproducible, and only short oligomers of up to 10-mers were produced conclusively in recent experiments, with the abundance rapidly decreasing with the oligomer length^13,18–20^. This trend is also consistent with the theoretical expectation for random adding of monomers (see below). An experimental difficulty is that aggregates may easily be mistaken for polymers, depending on detection methods^13,21^.

It is theoretically speculated that terminal ligation of oligomers may hierarchically produce further longer polymers^22^, but there is no experimental or quantitative demonstration of this starting from realistic prebiotic conditions. A report^23^ of experimental production of long polymers (>120 nt) by ligation has been subject to reproducibility and the aggregate/polymer discrimination problem^13,21,24^. A high concentration of oligomers is necessary for ligation to work efficiently, but this may be difficult because oligomer abundance rapidly decreases with oligomer length in polymerization by monomers, even if such a ligase activity exists.

If we consider only the conservative abiotic polymerization, i.e., statistically adding monomers, the probability of abiogenesis may be extremely low on a terrestrial planet. This case is not in contradiction with our existence on Earth, because we would find ourselves on a planet where abiogenesis happened. The life on Earth is believed to have descended from the single last universal common ancestor (LUCA) with no evidence for multiple abiogenesis events, and we do not know any life of a different origin in the universe. The emergence of life early in the history of Earth is often used to argue for a high abiogenesis rate, but an arbitrarily low rate cannot be robustly excluded^25–27^ because the chance probability *t*_*ab*_∕*t*_⊕_ ~ 0.1 is not negligible assuming a constant abiogenesis rate, where *t*_*ab*_ is the time of abiogenesis elapsed from the birth of Earth^28^, and *t*_⊕_ the present age of Earth. It may well be possible that early Earth was a more favorable environment for abiogenesis than present^29,30^. There may also be an anthropic selection effect favoring earlier abiogenesis on Earth, because an intelligent life must emerge before the increasing solar luminosity causes an end to Earth’s habitable state (estimated to be ~1 Gyr in future)^31^.

For the case of a low abiogenesis rate, the number of abiogenesis events is often considered in the Milky Way Galaxy containing about 10^11^ Sun-like stars^32^ or in the whole observable universe containing 10^22^ stars^33^ inside a spherical volume with a comoving radius of 46.3 Gly (or 13.8 Gly as a light travel time distance)^26,34^. However, the size of the observable universe is not related at all to its whole physical size. According to the widely accepted view of the inflationary cosmology^35–40^, the physical size of the universe created by an inflation event should be much larger, likely including more than 10^100^ stars (see below). In that case, even if the expected number of abiogenesis events is much less than unity in a volume size of the observable universe, it may still be consistent with our observations provided that abiogenesis is expected to occur somewhere in an inflationary universe.

The aim of this work is to examine this possibility quantitatively, assuming that the first biological RNA polymer was produced by randomly adding monomers. Koonin^41^ considered implications of the eternal inflation theory for the origin of life. In this scenario, most part of the universe inflates forever, self-reproducing many subregions that undergo a conventional inflation followed by a hot big-bang universe. Then an infinite number of stars and galaxies would be formed, and we expect emergence of life even if the abiogenesis probability is infinitely small. Though eternal inflation is a theoretically likely scenario^42^, it is difficult to confirm observationally, and a quantitative discussion is impossible. It is then interesting to ask if life can emerge within the homogeneous region in which we exist, assuming its minimal size necessary to explain observations. This work tries to give a quantitative answer to this question.

## The Size of the Universe Created by an Inflation Event

The observable universe is highly homogeneous and spatially flat on scales many orders of magnitude larger than the causally connected scale (horizon) in the early universe, which are called the horizon and flatness problems, and cannot be explained by the standard big bang cosmology. Cosmic inflation is currently the only widely accepted solution to these problems, and furthermore, it naturally generates scale-invariant quantum density fluctuations that serve as the seed of galaxy formation and the large scale structure in the present universe. Its prediction is in quantitative agreement with the observations of the cosmic microwave background radiation anisotropy, already constraining some theoretical models^43^.

There are many models and scenarios about how inflation occurred in the early universe^42^, but all of them consider an epoch of exponential expansion as *a* ∝ exp(*H*_*i*_ *t*_*i*_), where *a* is the scale factor of the universe, *H*_*i*_ the Hubble parameter at the time of inflation, and *t*_*i*_ the duration of inflation. If the inflation occurred at the energy scale of the grand unified theory of particle physics (10^16^ GeV), *H*_*i*_ would be about 10^37^ s^−1^. To solve the horizon and flatness problems, the *e*-folding number of inflation (*N*_*i*_ ≡ *H*_*i*_ *t*_*i*_) must be larger than^44,45^*N*_*i*,min_ ~ 60. If *N*_*i*_ = *N*_*i*,min_, a causal patch region expanded by the inflation has now the same size as the observable universe. It would be a fine tuning if the inflation duration is extremely close to the minimal value to solve the problems (i.e., *N*_*i*_ − *N*_*i*,min_ ≪ *N*_*i*,min_). Rather, we naturally expect *N*_*i*_ − *N*_*i*,min_ ≳ *N*_*i*,min_. If the inflation duration is twice (three times) as much as that required to solve the problems, the homogeneous universe should extend *e*^60^ (*e*^120^) times as much as the currently observable universe, which is 10^78^ (10^156^) times as large in volume, thus including about 10^100^ (10^178^) stars.

## Poissonian RNA Polymerization

Here we consider a cycle of RNA polymerization by randomly adding activated monomers to an oligomer as a Poisson process, taking an experiment on clay surfaces^18^ as a model case. Non-RNA nucleic acid analogues may have carried genetic information before the RNA world emerged^1^, but the formulations below can also be applied to such cases. Let *x*_*l*_ be the abundance of *l*-nt long oligomers. After the injection of activated monomers at the time of initialization (*t* = 0), evolution of *x*_*l*_ is described by the following differential equations:

1 $${\dot{x}}_{l+1}=\kappa \ {x}_{l}-\kappa \ {x}_{l+1}\ ,$$

where the dot denotes a time derivative. Here we assume that the coefficient *κ* (probability of a reaction with a monomer per unit time) does not depend on the oligomer length, which is approximately consistent with the trend found in the experiment^18^. We consider initial conditions of *x*_*l*_ = 0 for *l* ≥ 2, and *x*_1_ can be approximated to be constant in the early phase. The second term on the right hand side can be neglected when *x*_*l*+1_ ≪ *x*_*l*_. Solving the equations iteratively under these conditions, the abundance *x*_*l*_ at a time *t* is obtained as

2 $${x}_{l}=\frac{{p}_{r}^{l-1}}{(l-1)!}\ {x}_{1}\,,$$

where *p*_*r*_ ≡ *κ* *t* is the reaction probability with a monomer up to the time *t*. A similar result is obtained by considering the Poisson distribution with an expectation value of *p*_*r*_; the only difference is a factor of exp(−*p*_*r*_) that is not important at *p*_*r*_ ≲ 1. We should consider only the regime of *p*_*r*_ ≲ 1, because by the time *t* ~ *κ*^−1^, a significant fraction of activated monomers are lost by the reactions, and hence the approximation of constant *x*_1_ is no longer valid and efficient polymerization is not expected beyond this point. If activated monomers are lost earlier by some other processes (e.g. hydrolysis), *p*_*r*_ would be smaller than unity.

In RNA oligomerization on clay surfaces, the coefficient *κ* should be proportional to the concentration of activated monomers adsorbed on the clay surface. This clay-phase monomer concentration increases with that in aqueous phase, but according to the Langmuir adsorption isotherm, it saturates when the adsorbed monomer abundance reaches that of the exchangeable cations on clay surface. In the experiment^18^, montmorillonite has 0.8 mmol exchangeable cations per gram, and it starts to saturate at an aqueous monomer concentration of ~ 0.01 M (=mol/L). At the saturated clay-phase monomer concentration, the reaction rate is *κ* ~ 1 h^−1^, and thus *p*_*r*_ ~ 1 is reached within a few hours, which is much shorter than the hydrolysis time scale of activated monomers. Aqueous monomer concentration needs to be higher than a certain level to keep *κ* large enough for *p*_*r*_ ~ 1, and this may be achieved at some points during a cycle, for example, by variable amount of water expected in dry-wet cycles around warm little ponds^29^.

## Probability of an Active RNA Formation in the Universe

Let *l*_min_ be the minimum length of an RNA that needs to be abiotically formed for an emergence of life, and suppose that a *l*_min_-nt long, randomly polymerized RNA molecule acquires the necessary activity with a probability *P*_*ac*_ by a correct informational sequence of nucleotides. Once such an active polymer is produced, it proceeds to the stage of Darwinian evolution with a probability *P*_*ev*_, thus completing an abiogenesis process. Then we can calculate the number of abiogenesis events in a region of the universe containing N_*_ stars as

3 $${N}_{{\rm{life}}}={N}_{\ast }\ {f}_{pl}\ {t}_{d}\ {r}_{p}\ {P}_{ac}\ {P}_{ev}\ $$

where *f*_*pl*_ is the number of habitable planets per star, *t*_*d*_ the time during which abiotic RNA polymerization cycles continue, and *r*_*p*_ the production rate of *l*_min_-nt long RNA polymers on a planet. The production rate by the Poissonian process can be expressed using Eq. 2 in the previous section as

4 $${r}_{p}={N}_{m}\,\frac{{p}_{r}^{{l}_{min}}}{{l}_{min}!}\,{t}_{c}^{-1}$$

where *N*_*m*_ is the number of activated monomers participating in a cycle of polymerization on a planet, *t*_*c*_ the repeating time interval of polymerization cycles, and an approximation of *l*_min_ ~ *l*_min_ − 1 is used for simplicity. The baseline value of *p*_*r*_ is set to unity in the following analysis.

The probability *P*_*ac*_ can be expressed as

5 $${P}_{ac}=\frac{{N}_{ac}}{{N}_{nb}^{{l}_{min}}},$$

where *N*_*nb*_ is the number of nucleobase types participating in polymerization, and *N*_*ac*_ is the number of active sequences among all the possible sequences of a *l*_min_-nt long RNA polymer. We adopt *N*_*nb*_ = 4 as the baseline from RNA/DNA of life as we know it, but probably this is an underestimate for abiotic polymerization, because regioselectivity, homochiral selectivity, or any other reacting molecules that stop further polymerization would effectively increase *N*_*nb*_. The parameter *N*_*ac*_ is highly uncertain. Here we convert this parameter into Δ*l* (or *l*_eff_ ≡ *l*_min_ − Δ*l*) defined by the relation ${N}_{ac}\equiv {N}_{nb}^{\Delta l}$, so that

6 $${P}_{ac}=\frac{1}{{N}_{nb}^{{l}_{min}-\Delta l}}=\frac{1}{{N}_{nb}^{{l}_{{\rm{e}}{\rm{f}}{\rm{f}}}}.}$$

Considering an example with *l*_min_ = 40, there are 4^40^ ~ 10^24^ possible sequences of 40-mers, and perhaps *N*_*ac*_ = 10^4^ sequences out of them may have a replicase activity^8^, in this case Δ*l* = 6.6. Here we take Δ*l* = 0 as the baseline value, which is valid when Δ*l* ≪ *l*_min_.

Requiring *N*_life_ = 1 and taking a logarithm of Eq. 3, we find the number of stars necessary to expect at least one abiogenesis event in their planetary systems, as

7 $$2.3\,\mathrm{lg}{N}_{\ast }=\mathrm{ln}({l}_{min}!)-{l}_{min}\mathrm{ln}{p}_{r}+\,({l}_{min}-\Delta l)\,\mathrm{ln}{N}_{nb}-\mathrm{ln}C$$

where

8 $$C\equiv {f}_{pl}\ {N}_{m}\ {t}_{d}\ {t}_{c}^{-1}\ {P}_{ev}$$

and lg and ln are the common and natural logarithms, respectively. We need to determine the five parameters included in *C*. Obviously there are huge uncertainties, probably more than 10 orders of magnitude in total. However, these parameters appear only logarithmically, and we will find that these uncertainties hardly affect the main conclusions derived in this work.

We use *f*_*pl*_ = 0.1 as the baseline for the planet parameter^46^, which is the least uncertain among these, owing to the rapid development of exoplanet studies in recent years. The baseline for *t*_*d*_ is set to 0.5 Gyr as a plausible time scale from the birth of Earth to the abiogenesis^28^, and that for *t*_*c*_ is set to 1 yr supposing a seasonal cycle (e.g. ref. ^29^), though 1 day may also be reasonable for a day-night cycle. The parameter *P*_*ev*_ is highly uncertain, but *P*_*ev*_ = 1 is set as the baseline, which is optimistic but may not be unreasonable because a long RNA polymer assembled by the Poisson process would be rare and there would be no competitor or predator around it. Any other essential factors involved in the origin of life, e.g., encapsulation by membrane vesicle formation, may significantly reduce this parameter. It has been known that both RNA polymerization and vesicle assembly are accelerated on clay surfaces^1,4,14^.

The amount of monomers, *N*_*m*_, is probably the most uncertain parameter among the five in *C*. An upper limit may be estimated by the number of nucleotides in the present life on Earth, *N*_*m*_ = 7 × 10^37^ (3.7 × 10^16^ g in mass), which is estimated by the total biomass of 550 Gt-C (3.7 × 10^12^ wet ⋅ t)^47^ assuming that nucleic acids constitute 1% of the wet biomass. A rough amount of nucleobases delivered from space by meteorites can be estimated as follows (see ref. ^29^ for a more detailed modeling). The mass delivery rate of meteoroids from 4.5 to 4.0 Ga is 10^20−25^ kg/Gyr, and 0.1% of this mass belongs to meteoroids of a diameter 40–80 m, which efficiently deliver nucleobases avoiding melting or vaporization. Carbonaceous meteorites contain nucleobases with a mass fraction of 10^−7^, and they are deposited into warm little ponds which cover a fraction of 10^−6^ on the Earth surface. These nucleobases survive for 1 yr, i.e., a seasonal cycle before they are destroyed by UV radiation or seepage. Then we expect 10^20−25^ nucleobases (0.01–10^3^ g in mass) in the ponds. Instead, nucleobases may also be produced on Earth, and it would not be unreasonable to assume a similar nucleobase/carbon mass ratio to that found in carbonaceous meteorites (10^−5^). Assuming a carbon abundance similar to the present seawater, we expect 10^27^ nucleobases in the ponds assuming their depth to be 1 m. We then use *N*_*m*_ = 10^25^ as the baseline, though it could be wrong by many orders of magnitude, depending on various scenarios of nucleotide formation and their activation under prebiotic conditions. Using the baseline parameter values thus determined, we find ln*C* = 75.3.

## The Minimum RNA Length Versus the Universe Size

**Figure 1:**
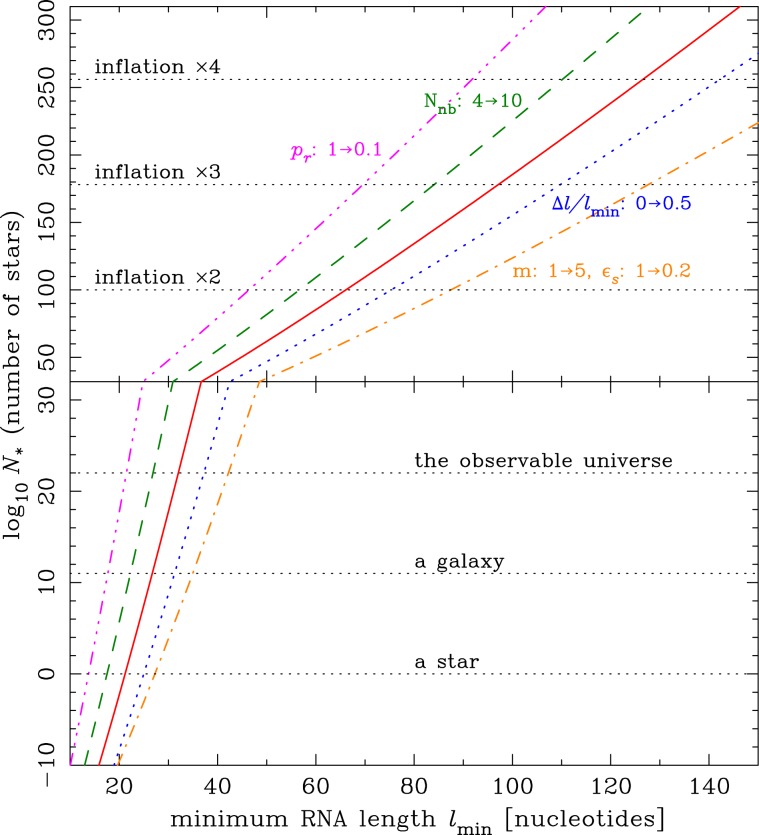
Logarithm of the number of stars necessary to expect at least one abiogenesis event (lgN_*_) versus the minimum RNA length required to show a biological activity leading to abiogenesis (*l*_min_). The difference between the top and bottom panels is just the scale of the vertical axis. Some important values of lgN_*_ are indicated by horizontal dotted lines; "inflation ×2” means the universe size when the inflation lasted twice as long as that required to make the observable universe homogeneous. The red solid curve is the relation using the baseline model parameter values, and other curves are when some of the model parameters are changed from the baseline values, as indicated in the figure.

Figure 1 shows lgN_*_ versus *l*_min_ for *N*_life_ = 1 calculated by Eq. 7. When the baseline parameter values are used, the minimum RNA length must be *l*_min_ = 21, 27 and 32 to expect one abiogenesis event for a survey of a single star (lgN_*_ = 0), a galaxy (lgN_*_ = 11), and the observable universe (lgN_*_ = 22), respectively. These *l*_min_ values are not sufficiently large compared with that (~40–100) required to expect an RNA replicase activity from a biological viewpoint, implying that abiogenesis is not easy even if we consider the entire volume of the observable universe. For *l*_min_ = 40 we find lgN_*_ = 39. If we try to reduce this to lgN_*_ = 22 or 0 for the same *l*_min_ by the uncertainty in *C*, this parameter needs to be increased by a factor of 10^17^ or 10^39^, respectively.

However, if we request one abiogenesis event somewhere in the whole physical volume created by an inflation, the chance of abiogenesis greatly increases. In a volume created by a twice, three, and four times as long inflation as that required to create the observable universe (lgN_*_ = 100, 178, and 256), *l*_min_ becomes 66, 97, and 127, respectively. These *l*_min_ lengths now allow us to expect a self-replicating activity of an RNA molecule. If an identical pair of RNA strands is required for abiogenesis, *l*_min_ should be effectively twice as large as each of the identical strands. Then an inflationary universe can produce a pair with a length of ~33–64 nt for each, and we can still expect a replicase activity. It is also possible that the inflation duration is even longer than the examples considered here.

Using the Stirling’s approximation, Eq. 7 can be written as

9 $$2.3\,\mathrm{lg}{N}_{\ast }\sim {l}_{min}\,(\mathrm{ln}{l}_{min}+\mathrm{ln}{N}_{nb}-1)-\mathrm{ln}C$$

when *p*_*r*_ = 1 and Δ*l* = 0. In the large limit of *l*_min_, ln*N*_*nb*_ − 1 can be neglected, and a useful approximated formula is:

10 $$\mathrm{lg}{N}_{\ast }\sim {l}_{min}\,\mathrm{lg}{l}_{min}-\mathrm{lg}C.$$

It should be noted that lgN_*_ changes only by 10 when a factor included in *C* is changed by 10 orders of magnitude; lgN_*_ changes from 167 to 177 for *l*_min_ = 100 for example, which hardly affects the main conclusion of this work.

Figure 1 also shows the lgN_*_-*l*_min_ relation when some model parameters are changed. The main results described above are not seriously changed when we change *N*_*nb*_ = 4 → 10 or Δ*l*∕*l*_min_ = 0 → 0.5. If we reduce *p*_*r*_ from 1 to 0.1, *l*_min_ is reduced from 66 to 46 for lgN_*_ = 100 (a twice as long inflation). A sufficient number of abiogenesis events may not be expected when *p*_*r*_ ≪ 1, even in the total volume of an inflationary universe.

A possibly important process is polymerization over multiple cycles. In polymerization on clay surfaces, inactive monomers and oligomers left from the previous cycle must be released from a clay surface for the next cycle to work, but a fraction of long oligomers may remain on the surface. Adding newly activated monomers to such oligomers over many cycles may be an efficient way to assemble a long polymer. Such a polymerization process may be limited by a time scale of RNA oligomer destruction, e.g., by hydrolysis or UV radiation during the dry phase. As a toy model to consider this, suppose that a fraction *ϵ*_*s*_ of oligomers survive to the next cycle. If polymerization of an oligomer continues over *m* cycles, the most efficient path to form a *l*_min_-nt polymer would be to repeat *m* times the process of adding *l*_min_∕*m* monomers. Then the polymer production rate *r*_*p*_ of Eq. 4 should be replaced by

11 $${r}_{p}={N}_{m}\,{[\frac{{p}_{r}^{{l}_{min}/m}}{({l}_{min}/m)!}]}^{m}\,{\epsilon }_{s}^{m}{(m{t}_{c})}^{-1}.$$

The result for *m* = 5 and *ϵ*_*s*_ = 0.2 is shown in Fig. 1 as an example, using the Gamma function for the factorial when *l*_min_/*m* is not an integer. In this case *l*_min_ becomes 42 for lgN_*_ = 22, implying a possibility that abiogenesis has occurred more than once inside the observable universe. Though *m* and *ϵ*_*s*_ are highly uncertain, this possibility should not be overlooked.

## Conclusions

It has been shown that the first RNA polymer with a replicase activity can be abiotically assembled by the most conservative polymerization process, i.e., random Poissonian adding of monomers, if we require that it occurs more than once somewhere in the physical volume of a universe created by an inflation, rather than inside the observable universe for us. This gives a simple solution to the abiotic polymerization problem to initiate the RNA world. Equation 7 relates two quantities on vastly difference scales: lgN_*_ on an astronomical scale and *l*_min_ on a biologically microscopic scale, and uncertainties of other parameters are not important because most of them appear logarithmically. This reminds us of an ouroboros.

The result of this work may also give an explanation for the homochirality of life. Even if activated monomers supplied to the polymerization cycle are a racemic mixture, life emerging from them would be homochiral, if homochirality is a necessary requirement for an RNA polymer to show biological activities. Simply it needs more time or volume for a homochiral polymer to be assembled by random polymerization, with *N*_*nb*_ twice as large as when ignoring chirality. As shown in Fig. 1, change of *N*_*nb*_ by a factor of two does not seriously affect the expected number of abiogenesis events in an inflationary universe.

On the other hand, the expected number of abiogenesis events is much smaller than unity when we observe a star, a galaxy, or even the whole observable universe. This gives an explanation to the Fermi’s paradox. The observable universe is just a tiny part, whose volume is likely smaller than 1∕10^78^ of the whole universe created by an inflation, and there is no strong reason to expect more than one abiogenesis event in such a small region. Even if Earth is the only planet that harbors life inside the observable universe, it does not contradict the Copernican principle, because life would have emerged on countless planets in the whole inflationary universe in which we exist.

In the picture presented here, the probability of finding biosignatures from planets or satellites in the Solar System or from exoplanets is negligibly small, unless we consider interplanetary or interstellar traveling of microorganisms^48,49^. It should be noted, however, that the case of a high abiogenesis rate (*N*_life_ ≳ 1 for N_*_ = 1) cannot be excluded by this work, because we assumed that abiotic RNA polymerization occurs only by the random Poisson process of adding monomers. Potential roles of much more efficient processes on the origin of life, such as non-linear auto- or cross-catalytic reactions, have been studied theoretically^50^, though it is highly uncertain whether such processes really worked in realistic prebiotic conditions. If organisms having a different origin from those on Earth are found in future, it would suggest that such a mechanism is working at abiogenesis to reduce *l*_min_. Although this possibility should not be excluded, what is shown by this work is that such a hypothetical process is not necessary if we request abiogenesis events to occur somewhere in an inflationary universe.

It is also worth pointing out that, in the lgN_*_-*l*_min_ relation for *N*_life_ = 1, lgN_*_ rapidly increases from 0 (a star) to 22 (the observable universe) in a short range of *l*_min_ = 21–32. Even if a non-linear process is working at some stages, the initial polymerization is likely statistical and random as considered here. Then it would be an extreme fine tuning if a biological parameter *l*_min_ is just close to the value corresponding to *N*_life_ ~ 1 for a star (N_*_ = 1). Rather, *N*_life_ ≫ 1 or *N*_life_ ≪ 1 is much more likely when we observe just one planetary system. As we have argued, the case of *N*_life_ ≪ 1 is not in contradiction with observations, but the opposite case may be in tension with the lack of evidence for multiple abiogenesis events in the history of Earth or in laboratories.

A fundamental assumption in this work is that an abiotically assembled RNA polymer acquires a self-replicating ability if it is sufficiently long and has a correct nucleotide sequence. This may be rather trivial under the physical laws ruling this universe, because we know that ribozymes are actually working in life and can also be produced by *in vitro* experiments. This work considered only a single homogeneous region in the universe created by an inflation event, obeying the same physical laws that we observe. However, the multiverse hypothesis^51^ implies existence of other universes created by different inflation events, in which physical laws may be different from ours. A theoretically intriguing question is whether a chemical RNA-like long polymer is easily formed to contain information and show biological activities eventually leading to higher organisms, when physical laws are arbitrarily made, e.g., by random choices of fundamental physical constants. Perhaps this may be the ultimate mystery regarding the origin of life, which is, of course, far beyond the scope of this work.
